# Comparative Evaluation of Hydrothermal Carbonization and Low Temperature Pyrolysis of *Eucommia ulmoides* Oliver for the Production of Solid Biofuel

**DOI:** 10.1038/s41598-019-38849-4

**Published:** 2019-04-02

**Authors:** Yajun Wang, Ling Qiu, Mingqiang Zhu, Guotao Sun, Tianle Zhang, Kang Kang

**Affiliations:** 1 College of Mechanical and Electronic Engineering, Northwest A&F University, Yangling, 712100 China; 2 Northwest Research Center for Rural Renewable Energy Exploitation and Utilization, Ministry of Agriculture, Yangling, 712100 China

## Abstract

This study evaluates the feasibility of two thermal pretreatments including hydrothermal carbonization (HTC) and low temperature pyrolysis (LTP) on the production of *Eucommia ulmoides* biochar. The waste wood of *Eucommia ulmoides* Oliver was pretreated and characterized for fuel applications. The results confirm that both LTP and HTC are promising processes for improving fuel properties. However, for the same char yield, the required temperature for HTC is lower than LTP, as the char yields of H_200_ and L_300_ were quite close (66.50% vs. 66.74%). The surface morphology is significantly different between the pyrolytic carbon and the hydrochar. In addition, it was found that the H/C and O/C ratios of H_300_ were 0.82 and 0.21, respectively, and the H/C and O/C ratios of L_340_ were 0.77 and 0.22, respectively. They were similar to that of sub-bituminous. Moreover, under the same reaction temperature, hydrochar showed better grindability, hydrophobicity, and reduction in inorganic content. Comparing the integrated combustion characteristic index (*S*), LTP process had the better performance within the lower temperature under 220 °C while HTC process performed better at temperature higher than 300 °C. The results reveal that HTC has the potential to produce solid carbonized products with better fuel quality.

## Introduction

Global warming and excessive fossil energy consumption call for the development of bioenergy. Bioenergy can be obtained from forestry residues, agriculture wastes, energy crops, manures, and municipal wastes^1–3^. Among these renewable options, lignocellulosic biomass is the only carbon-neutral energy resource which covers the wide range of forestry residues, agriculture wastes,and energy crops^4–6^. Intensive cultivation of energy crops will occupy the arable land, while the disposal of agricultural and forestry residues is in the rigid demand by agriculture and industry. Although a tremendous amount of forestry residues which are approximately 37 million m^3^ are produced annually in China^7^, the direct application of these biomass resource suffers from their low energy density, high moisture content, and high transportation cost^8^. Therefore, in order to overcome the issues mentioned above, these biomass resources have to be pretreated to improve their fuel quality^9^.

Recent researches interest in using the solid carbonized products as a substitute to coal has gained much attention^10–13^. Solid carbonized products have many advantages over conventional fossils fuels. First, biochar is produced from biomass, which is renewable and abundant worldwide. In contrast, fossil energy is limited, so the search for alternatives to fossil energy is critical^14^. Secondly, emission of greenhouse gases from the combustion of coal and the accompanying environmental problems remain the global challenges. The utilization of carbon-neutral biochar will be one of the main ways to alleviate this issue. In addition, another advantage of biochar over fossils lies in its low sulfur and nitrogen contents which give it great potential to reduce the pollution^15^. Low temperature pyrolysis (LTP) and hydrothermal carbonization are two effective thermal chemistry approaches to improve fuel quality to accommodate coal-fired furnace^16,17^. LTP is a traditional thermal pretreatment process to improve physicochemical properties. During LTP, biomass is heated under oxygen-free atmosphere with low heating rate and the main target product which is pyrolytic char is generated^18^. LTP technology is also applied for biochar production for soil additives^19^. It was also used for improving the fuel property of biomass which was also known as torrefaction, as a treatment of biomass before combustion processes^20^. HTC process was first reported in 1913 by Bergius and it has received wide attention in China in recent years^21^. HTC process, also referred to wet torrefaction, is usually performed in subcritical water at the temperature which is significantly lower than LTP. The raw material is heated in a confined atmosphere under the autogenous pressure^22^. Compared with HTC, the advantage of LTP is that the process is relatively simple; the technology is more mature and easy to industrialize. However, HTC has its own advantages. For example, since the carbonization reaction is carried out in water, no drying process is required for HTC. In addition, HTC can wash away the inorganic elemental compositions into the liquid phase and reduce the ash content^23^. The LTP and HTC can improve the fuel quality including increased fixed carbon content, reduced moisture content, improved hydrophobic and grindability of the raw biomass. There are many essential reaction parameters for both processes, such as reaction time, heating rate, and temperature. Although these parameters have been observed to influence the properties of hydrochar and pyrolytic carbon, the reaction temperature was reported to be the most critical parameter^24^.

Previous research has compared these two processes on various forestry and agricultural wastes. Harpreet Singh Kambo used miscanthus as raw material, comparative analyzed the impact of two processes on the solid product^9^. In the research of Zhengang Liu, coconut fibers and eucalyptus leaves were used to compare the process kinetics and combustion performance^25^.These researches are rich in literature. However, very few studies have paid attention to the comparative assessment of *eucommia ulmoides* for producing solid fuel via LTP and HTC.

*Eucommia ulmoides* Oliver is a deciduous plant species which originated from China. There are nearly three hundred and fifty thousand hectares in China, accounting for more than 99% of the world’s total resources. In Asia, its male flowers, bark, and leaves can be produced as a health supplement and Chinese herbal medicines^26^. At present, the application of *Eucommia ulmoides* has grown from a single medicinal extended to many important areas, such as *Eucommia* rubber, ecological protection, communications, and green farming industry, and that means it will get a larger planting area in future. Regrettably, unlike the male flowers, bark, and leaves, *Eucommia* trunk and branches have not been ever effectively utilized. Recently, the research of *Eucommia* waste mainly focused on fractionation and xylooligosaccharides production^27,28^. However, the research which regards its utilization as solid fuel by-product is still vacant.

Above all, the objective of this study is to comparatively evaluate LTP and HTC for the conversion of *Eucommia ulmoides* Oliver to chars by (1) investigating the HHV, product yield, and proximate analysis to understand the basic properties of chars. (2) analyzing the changes in organic elements and functional groups. (3) evaluating the fuel qualities of chars by inorganic element detection and combustion analysis. Moreover, scanning electron microscopy (SEM) was used for tracking the surface morphological changes of the chars.

## Material and Methods

### Materials

The trunk and branches of *Eucommia ulmoides* Oliver (without bark or leaves) were collected in Yangling, China. The sample prepared in powder form was sieved and the fraction with particles sizes between 0.42 mm–0.84 mm (40-20 mesh) was used for analysis. Before characterization, the samples were dried for 24 h at 105 ± 5 °C in an oven for the removal of moisture.

### Experimental setup

#### Hydrothermal carbonization experiments

A laboratory scale 500 ml reactor (FCF, Pengyi, China) was used for the hydrothermal carbonization. The reactor consists of a reactor body, a heater, and a condenser which was operated under nitrogen. 15 g sample was mixed with 225 ml deionized water and loaded into the reactor. The components within the reactor were vigorously mixed using an agitator rotating at 100 rpm. The operating temperatures ranged from 180 °C to 320 °C with 20 °C temperature intervals and the holding time was set as 30 min.

#### Low temperature pyrolysis experiments

The traditional low temperature pyrolysis (LTP) was conducted in a tubular furnace with the scale of 80 mm diameter and 1000 mm length (TF1000-80, ACX, China). 15 g of the sample was loaded into a corundum boat which was then placed in a tubular furnace and nitrogen with the 50 ml/min flow was introduced to maintain an inert atmosphere in the system. The final reaction temperature of low temperature pyrolysis (LTP) was set as 180–440 °C with 20 °C temperature intervals. All the samples were heated to the final temperatures at the heating rate of 10 °C/min and heat preservation for 30 min.

### Characterization methods

Proximate analysis was performed based on ASTM 1762-84 for the analysis of fixed carbon, volatile matter and ash. The definitions of biochar yield and fuel rate presented in this study are as follows^21^:

1 $${Biochar}\,{yield}=\frac{Mass\,of\,the\,biochar}{Mass\,of\,the\,raw\,biomass}\times 100 \% $$

2 $$Fuel\,rate=\frac{Content\,of\,fixed\,carbon}{Content\,of\,volatile\,matter}\times 100 \% $$

The higher heating value (HHV) of the samples was measured with an oxygen bomb calorimeter (ZDHW-9000, HK, China). A scanning electron microscope (TM 3030, Hitachi, Japan) was used to study the surface morphological changes of chars. Prior to analysis, <1 mg of samples were located on an adhesive sticker on an aluminum stub. Scanning electron images were then obtained with an incident electron beam at 5 kV at two different magnification ratios.

The content of C, H and N were carried out using elemental analyzer (1108CHN, Fisons, USA) and oxygen was calculated by difference. Cellulose analyzer (A200i, ANKOM, USA) was used to determine the contents of neutral detergent fiber (NDF), acid detergent fiber (ADF) and acid detergent lignin (ADL). The content of hemicellulose, cellulose and lignin are calculated as follows:

3 $$Content\,of\,Hemicellulose\,( \% )=NDF( \% )-ADF( \% )3NDF( \% )$$

4 $$Content\,of\,Cellulose\,( \% )=ADF( \% )3NDF( \% )-ADL( \% )3ADF( \% )3NDF( \% )$$

5 $$Content\,of\,Lignin/Aromatic\,compound\,( \% )=ADL( \% )-Ash( \% )$$

The infrared spectra were recorded with an FT-IR (Nicolet iS10, Thermo Scientific, USA) with the scanned area of 500–4000 cm^−1^. After deducting the atmospheric background, each sample was scanned 16 times. In addition, selected samples were analyzed for the inorganic elemental composition. After microwave digested with nitric acid in closed vessels, treated samples were brought to volume with ultra-pure water. The clear extract supernatant was measured by inductively coupled plasma mass spectrometry (iCAP-Qc, Thermo Scientific, USA).

A thermo-balance TGA (TGA/DSC, METTLER TOLEDO, USA) was used for evaluation of combustion properties of the chars. Experiments were conducted at an oxygen atmosphere, using temperatures ranging from room temperature to 800 °C with a heating rate of 10 °C/min and an oxygen flux of 20 mL/min. The moisture absorption behavior of the chars was explored in a constant temperature and humidity incubator (Model MD1400, Snijders, Netherlands). Before the test, the samples were dried in an oven at 105 ± 5 °C for 24 h, and then put into the incubator at the temperature of 30 °C and the constant relative humidity of 70%. They were measured every 30 min for the first 4 h, followed by every 120 min for the next 6 h.

## Results and Discussion

### Effect of process on the basic property of chars

#### Effect of HTC and LTP on char yield, HHV and energy yield

**Figure 1:**
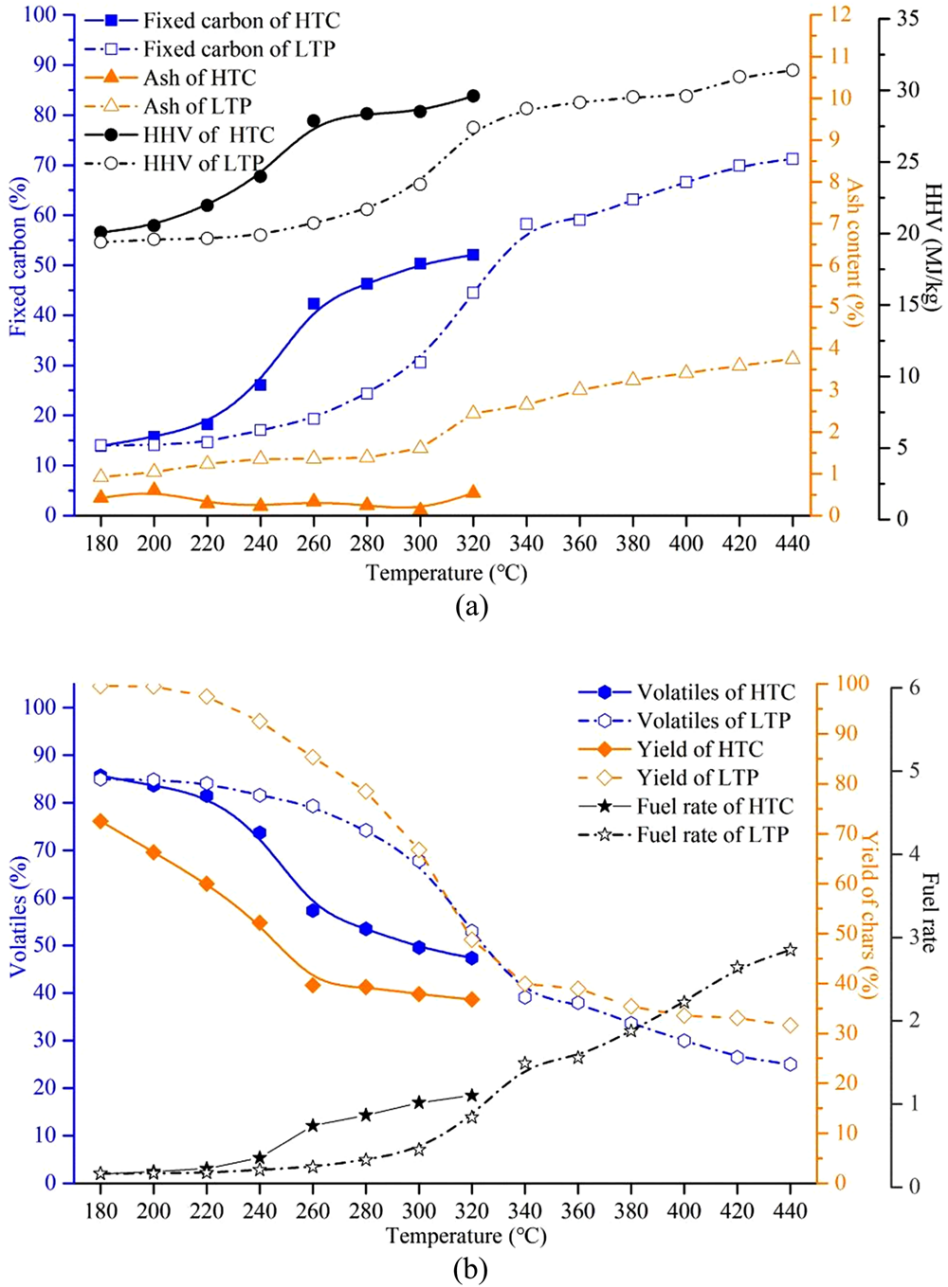
Proximate analysis, HHV, product yield and fuel rate of samples.

The main objective of this work was to compare the difference of chars produced via LTP and HTC in terms of their product yield and properties. For simplifying, char samples were named as following the format such as “Process _Temperature_” (e.g., H_220_ stands for hydrothermal carbonization conducted at 220 °C, whereas L_220_ stands for low temperature pyrolysis conducted at 220 °C). The proximate analysis, HHV, product yield and fuel rate are summarized in Fig. 1(a,b).

Although processed under the same reaction temperature and holding time, a higher yield of chars was observsevd with LTP. The downtrend of hydrochar yields lowed down once the reaction temperature of HTC exceeded 260 °C, while this phenomenon occurred near 340 °C in the LTP process. The results indicate that cellulose degraded significantly before 260 °C by HTC and 340 °C by LTP. It is similar to the previous study^9^. Regardless of the processes, the fixed carbon content increased, and the volatiles content declined with the increments of reaction temperature. For example, H_320_ has 52.10% fixed carbon and 47.35% volatiles. L_440_ has 71.25% fixed carbon and 25.00% volatiles. Compared to raw materials (13.56% fixed carbon, 85.47% volatiles), they both changed significantly.

In the LTP process, the ash content increased with increase in temperature as a result of the release of volatiles. Interestingly, the ash content of HTC did not show the same growth trend. Specifically, under the same reaction temperature, the ash content in HTC experiments was significantly lower than that of the LTP and fluctuated between 0.13% and 0.61%. For instance, even maximum ash content (0.61%) of HTC which was observed with H_200_ was much smaller than that of the L_200_ (1.05%). It could be explained by the dual effects by elution and accumulation of the ash. As is reported in Fig. 1(a), regarding the ash content, the ash removed by subcritical water is greater than its accumulation in chars.

The change of HHV versus temperature was similar to that of the fixed carbon. It proves that the content of fixed carbon is a major factor determining the HHV. In this study, the maximum HHV of the chars from HTC reached 29.61 MJ/kg (H_320_), and the maximum HHV of the chars from LTP reached 31.40 MJ/kg (L_440_). In the temperature range of 180–320 °C, the fuel rate of both HTC and LTP significantly increased from 0.16 to 1.10 and 0.84, respectively. The fuel rate of hydrothermal carbon has been higher at lower temperatures, such as H_260_ which had a fuel rate of 0.74, while the L_260_ has fuel rate of 0.24. In comparison, at the higher reaction temperature, chars from LTP had a higher fuel rate. For example, the fuel rate of L_440_ reached 2.85.

With the results from the proximate analysis, HHV as well as char yield, it was found that regardless the process of HTV or LTP, the rising tendency of fixed carbon content was highly consistent with that of HHV, both the tendencies of volatiles and char yield were similar. In addition, the char yields of H_200_ and L_300_ were quite similar (66.50% and 66.74%, respectively). While the chars from L_300_ has better basic fuel characteristics with 30.59% fixed carbon content (which was 15.71% of H_200_), 23.43 MJ/kg HHV (which was 20.54 MJ/kg of H_200_) and 0.45 fuel rate (which was 0.19 of H_200_). The HHV of H_240_ was closed to that of the L_300_ (23.99 MJ/kg and 23.43 MJ/kg), and both were higher than that of the standard lignite (23.00 MJ/kg). As was shown in Table 1, H_240_ and L_300_ have extremely similar composition of elements which could explain the similarity of HHV. However, H_240_ has lower fixed carbon content (26.07% to 30.59% of L_300_) and higher volatiles content (73.70% to 67.80% of L_300_). It shows that compared with L_300,_ H_240_ has stronger ignition performance and the higher heat release in the same heat production condition. The HHV of H_300_ and L_340_ were similar (28.52 MJ/kg and 28.73 MJ/kg) and reached the HHV of bituminous which was 27.17 MJ/kg^29^. In the comparison of elements, H_300_ has more C content which was 73.03% (65.59% of L_340_) and less O content which was 20.61% (28.05% of L_340_). In addition, a group of chars samples which were H_220_ and L_220_ under the same reaction temperature were selected for process comparison. It was found that H_220_ has slightly higher HHV and fuel rate but has lower chars yield. The characteristics compared to the latter are based on these groups.

**Table 1:** Elemental and fiber analysis of selected samples.

	Elemental analysis (wt%)			Fiber analysis (wt%)		
	C	H	O	Hemicellulose (%)	Cellulose (%)	Lignin/aromatic compound (%)
Raw	47.03	6.07	45.54	21.73	34.04	40.17
L_220_	48.97	5.93	44.02	21.33	36.45	40.65
L_300_	57.26	5.54	34.49	2.91	43.06	51.70
L_340_	65.59	4.59	28.05	2.06	30.28	59.52
H_200_	51.26	6.03	42.33	1.53	40.92	42.78
H_220_	53.03	5.94	40.66	1.52	40.27	43.43
H_240_	57.73	5.65	35.85	1.10	34.21	47.87
H_300_	73.03	5.01	20.61	1.01	22.86	51.74

*The results are in air dry basis.

#### SEM analysis

**Figure 2:**
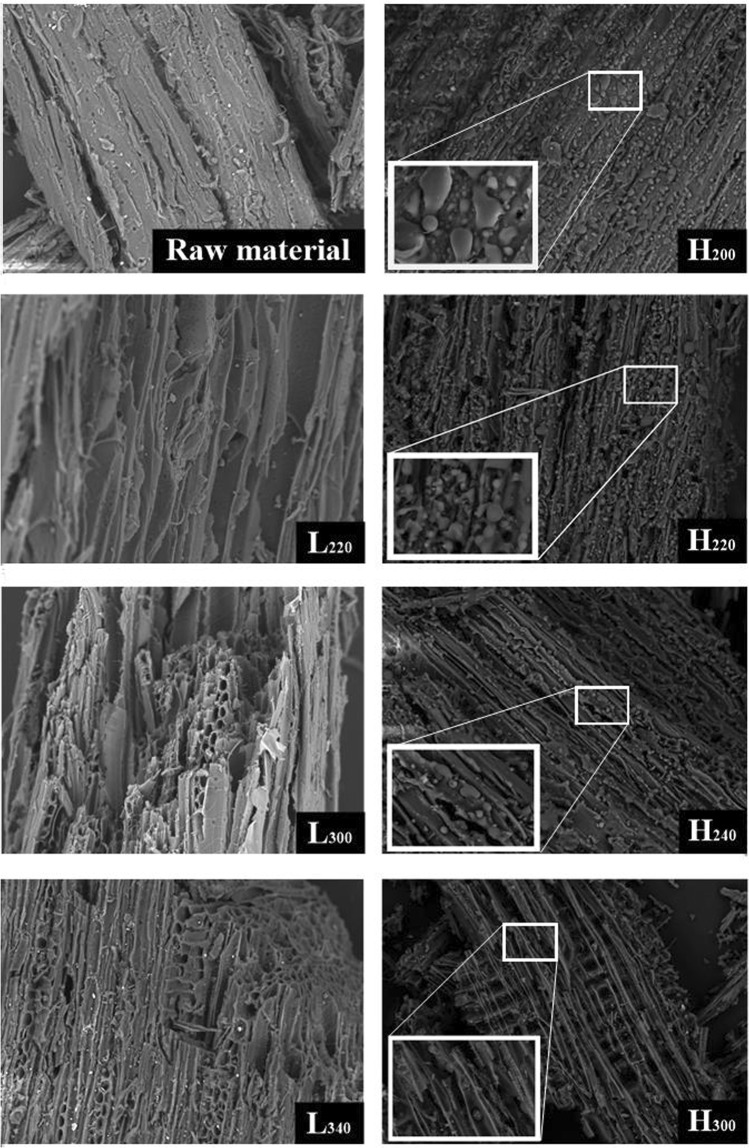
SEM images of chars prepared via HTC and LTP.

A close inspection of the raw material and selected samples was performed using scanning electron microscopy (SEM) technique. Images shown in Fig. 2 reveals different transformations in the morphologies of the hydrochars versus the pyrolytic carbon. As the figure shows, the structure of the raw material is less porous than the char samples of HTC and LTP. In the image of H_200_, H_220_ and H_240_, the hydrochars contained aggregates of spherical microparticles. These microparticles are originated from the decomposition of cellulose, and the subsequent precipitation and growth into spheres^30^. However, in the image of H_300_, these spherical microparticles almost disappeared completely. This is most likely due to that they have been broken down into small molecules and was dissolved in the liquid phase under this temperature. Comparing the images of L_220_, L_300_, and L_340_, it was found that higher reaction temperatures of LTP contributed to the formation of pores and resulted in increased roughness on the biomass surface. By the result of char yield and SEM image from Fig. 2 (H_220_ and L_220_, H_300_ and L_300_), it could be concluded that the lignocellulosic structure of the HTC was decomposed much more significantly than LTP under the same reaction temperature.

### Effect of HTC and LTP on the physicochemical properties of chars

#### Elemental analysis and fiber analysis

**Figure 3:**
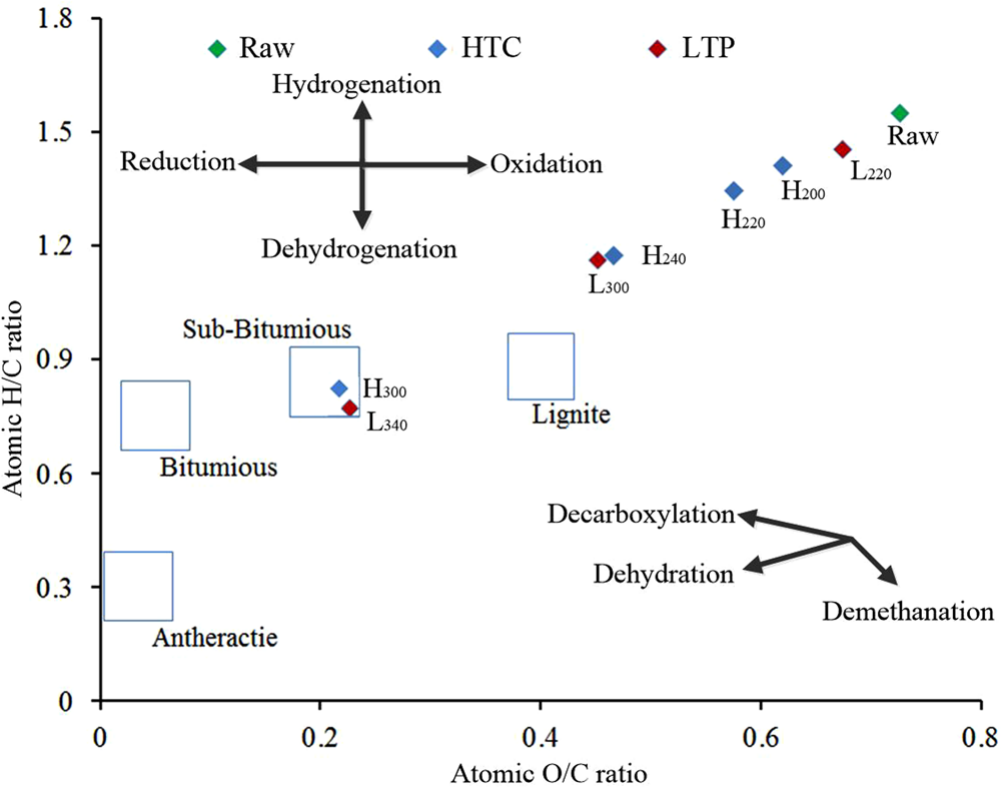
Van Krevelen diagram of selected samples.

As shown by the results of the elemental analysis in Table 1, regardless of the process of HTC or LTP, carbonization caused the aggregation of carbon element and the releasing of hydrogen and oxygen. To analysze the variation in elemental composition, the ratios of hydrogen to carbon (H/C) and oxygen to carbon (O/C) of selected samples were ploted in Fig. 3 in the form of “Van Krevelen diagram”^31^. The diagram confirms that both of the HTC and LTP had a significant effect on the elemental composition of the products. Specifically, in Fig. 3, after the treatment of HTC, when the reaction temperature increased to 300 °C, the atomic H/C and O/C ratios dropped from 1.55 and 0.73 to 0.82 and 0.21, repectively. The atomic H/C and O/C ratios of L_340_ were 0.77 and 0.22, respectively. Four typical coals such as anthracite, bituminous, sub-bituminous, and lignite were used for comparision with the prepared chars. It was found that the H/C and O/C ratios of H_300_ and L_340_ were in the sub-bituminous region. In addition, with the increasing of temperature, the H/C and O/C atomic ratios of the samples decreased and became closed to that of coal.

Comparing the fiber analysis results of H_220_ and L_220_ (Table 1), it is obvious that most of the hemicellulose was removed at a lower temperature in the HTC process. However, the hemicellulose content in L_220_ is very close to that of the feedstock, indicating that most of the hemicellulose in pyrolytic carbon was not degraded in 220 °C via LTP process. This also explains the fact that the yield of hydrothermal carbon is generally higher than that of pyrolytic carbon at the same temperature (Fig. 1b). Therefore, it was considered that the degradation of hemicellose is the critical factor which influenced the results of the proximate analysis, char yield, and HHV in lower temperature region. Regadless of the processes, the content of cellulose was always experienced an initial stage of ascending and a subsequent desending. For example, the content of cellulose (40.92%) in H_200_ was higher than that of the raw material (34.04%) and H_240_ (34.21). For the LTP treatment, the content was 43.06% in L_300_ was higher than that of L_220_ (36.45%) and L_340_ (30.28%). Above all, it can be concluded that the increasing of cellolose content in lower temperature region was mainly due to the removal of hemicellulose. The subsequent decrease in the content of cellulose is mainly due to the decomposition of the cellulose.

#### FT-IR spectra of chars

**Figure 4:**
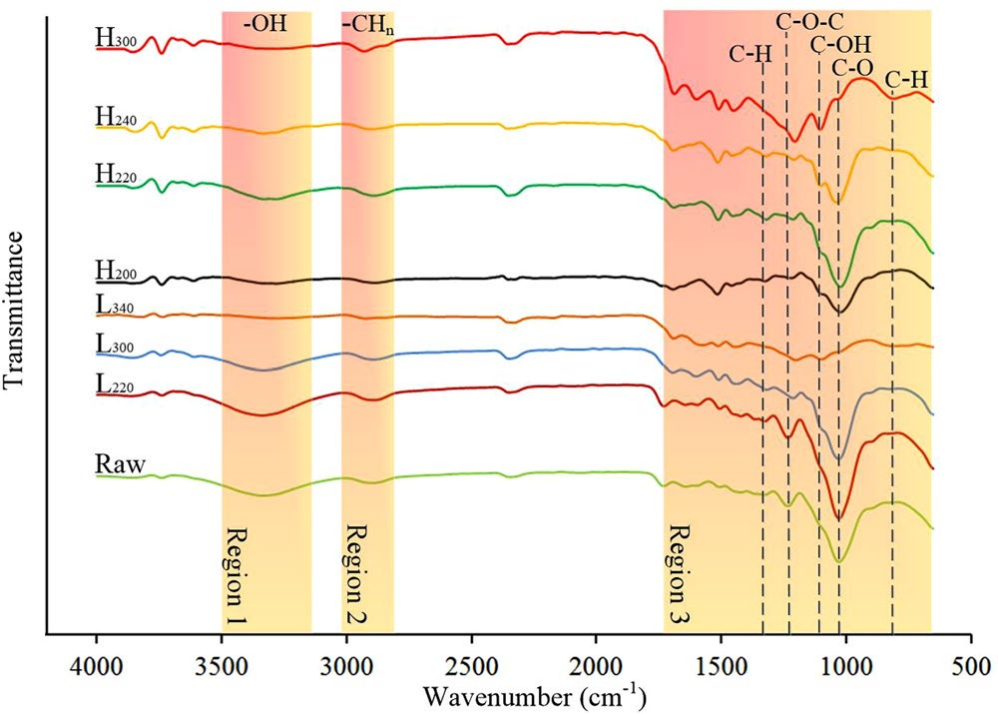
FT-IR spectra of selected samples.

The FT-IR spectra of the selected samples are shown in Fig. 4. It could be observed that the raw materials and treated samples showed similar spectral patterns, however, the intensities of infrared absorption peaks are differnt. Besides, it is worth noticing that due to the presence of CO_2_ during the measurement, a peak around 2300–2400 cm^−1^ was observed. In region 1, due to the vibration of –OH stretching, the peak between 3200 cm^−1^ and 3600 cm^−1^ can be found. It is obvious that there was a decrease in the intensity of –OH peak with the increase in the reaction temperature regardless of HTC or LTP^32^. The peaks between 2700 and 3000 cm^−1^ represent the aliphatic groups of CH, CH_2_ and CH_3_, respectively^33^. The content of aliphatic compounds was increased by the HTC treatment as slight incerase of the peak intensity was found, while it was not obvious with the LTP process. Various functional groups were identified in region 3. The peak around 1370 cm^−1^ represents the C–H stretching vibration and deforming vibration of cellulose and hemicellulose. With the rising reaction temperature, the dehydration and depolymerization of cellulose and hemicellulose caused the decrease in peak strength. The peaks between 1230 cm^−1^ and 1257 cm^−1^ are the characteristics of C–O–C vibrations in the cellulose and hemicellulose. These peaks shrinked with the increase in temperature by both HTC and LTP processes, which proved the decomposing of cellulose and hemicellulose^34^. In addition, compared with the raw material, new absorption peaks appeared at 1000–1100 cm^−1^ prove the presence of C–OH in alcohol groups^33^. The peak appeared at 1025 cm^−1^ (1000–1033 cm^−1^,C–O in methoxy) decreased, because the C–O band was broken due to the decarboxylation reation. It indicated that lignin was degraded during HTC and LTP processes. The peaks appeared at 765 cm^−1^ could be assigned to the C–H group in substituted aryl, which was observed in the samples treated with higher reaction temperatures such as H_300_ and L_340_. It means the aromatization of lignin was more pronounced in H_300_ and L_340_. Comparing H_300_ and L_300_ which were prepared under the same reaction temperature, it was found that H_300_ showed weaker absorption peaks of –OH (3200–3600 cm^−1^) and C–H which at 1370 cm^−1^, while had stronger absorption peaks of C–H at 765 cm^−1^. It shows the HTC process enabled stronger carbonization at the same reaction temperature.

### Effect of process operating conditions on the fuel properties of chars

#### Major ash content related metals analysis

The ash content and composition of feedstock are important indexes which need to be assessed for fuel utilization. Most lignocellulosic biomasses are rich in inorganic elements which are the main components of ash in raw materials and chars. These inorganic elements, including magnesium (Mg), sulfur (S), calcium (Ca), manganese (Mn), copper (Cu), zinc (Zn) and iron (Fe), often existed in the biomass in the oxidized forms, MgO, SO_3_, CaO, Mn_3_O_4_, CuO and Fe_2_O_3_, respectively. These can cause slagging, fouling and corrosion of boiler. It has shown in Fig. 1(a) that the ash contents in HTC samples were significantly lower than that of the LTP samples prepared under the same reaction temperature and the values are fairly low (0.13–0.61%), which is due to the leaching of inorganic compositions. The different behavior observed for the LTP process is the consequence of the concentration effect. Table 2 shows the effect of HTC and LTP processes on the inorganic composition yields of hydrochar and pyrolytic carbon. The results suggest that HTC and LTP are different in the mechanism of yielding inorganic elements. It is evident that the HTC process removed the considerate amount of the inorganic components (26–90%) from the raw feedstock, especially magnesium, calcium and manganese. In comparison, the contents of inorganic elements in pyrolytic carbon were higher than that of the raw material. With the increase in reaction temperature, the volatiles was separated from the sample and the inorganic elements were hence concentrated in the produced chars. The observations are in coherence with the finding reported in the literature^9,24,25^.

**Table 2:** Yields of inorganic elements in selected char samples.

Metal	Inorganic yield (%)						
	L_220_	L_300_	L_340_	H_200_	H_220_	H_240_	H_300_
Magnesium	+1.02	+36.52	+108.24	−71.21	−74.62	−81.72	−82.71
Sulfur	−21.79	−11.78	+1.91	−26.00	−43.92	−58.10	−65.94
Calcium	+11.61	+35.63	+84.62	−66.21	−78.73	−80.18	−82.92
Manganese	−38.5	+79.39	+93.58	−81.10	−90.38	−86.86	−86.28
Copper	+36.09	+22.16	+36.42	5.45	21.14	12.76	−6.43
Zinc	−8.42	+15.51	+26.83	−43.23	−59.19	−49.03	−50.13
iron	+15.21	−14.78	+54.89	−64.89	−64.29	−57.51	−44.91

^*^The yields of inorganic elements in all the char samples were compared to the raw materials. Taking L_220_ as an example: Compared with raw materials, Magnesium detected in L_220_ increased by 1.02% while the Sulfur decreased by 21.79%.

#### Analysis of combustion characteristics

**Figure 5:**
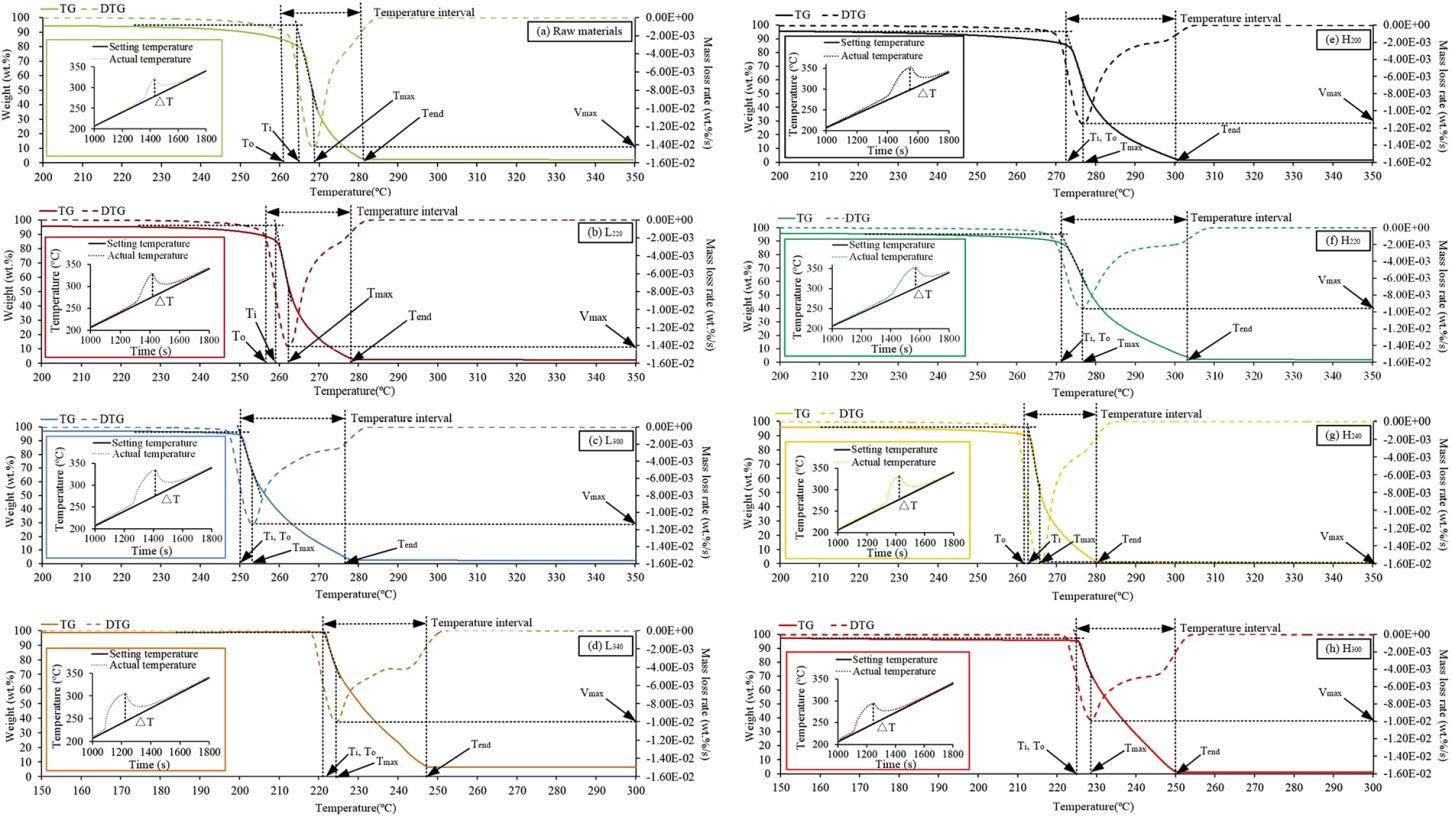
TG and DTG curves of selected samples (**a**, Raw materials; **b**, L_200_; **c**, L_300_; **d**, L_340_; **e**, H_200_; **f**, H_220_; **g** H_240_; **h** H_300_).

To evaluate the effects of HTC and LTP processes on the combustion properties, the samples were subjected to thermo-gravimetric (TG) analysis under an oxidizing atmosphere. The TG and DTG curves of raw materials, pyrolytic carbon and hydrochar are shown in Fig. 5(a–h), respectively. For clarity, the regions at temperatures lower than 200 °C (L_340_ and H_300_ were lower than 150 °C) and higher than 350 °C (L_340_ and H_300_ were higher than 300 °C) are excluded. Table 3 presents the corresponding combustion parametres for selected samples obtained from TG and DTG analysis. *Ti* (ignition temperature) means the lowest temperature of the combustion reaction. Temperature interval was defined as the range where the sample mass decreasing rates are above 1%/°C. *V*_*max*_ and *T*_*max*_ were the maximum value of weight loss rate and its corresponding temperature. Δ*T* was the maximum value of the temperature difference between actual temperature and set temperature due to the burning of the sample. In the pyrolytic carbons, it was observed that the ignition temperature of the sample decreased from 265 °C to 221 °C when the pyrolysis temperature increased. Hydrothermal carbon also showed a similar trend in the change of ignition points. Generally, in a wider pyrolysis temperature range (<800 °C), the ignition point of the fuel shifted to a higher temperature range with the deepening degree of carbonization and decreasing content of volatiles. However, selected samples did not show the similar trend. It implies that the most important factor affecting the ignition is not the content of volatiles in the lower temperature range.

**Table 3:** Ombustion characteristic of selected samples.

	Ti (°C)	Temperature interval (°C)	T_max_ (°C)	V_max_ (wt%/s)	ΔT (°C)
Raw	265	261–281	268	−1.43E-02	45
L_220_	259	257–278	263	−1.41E-02	53
L_300_	251	250–277	253	−1.14E-02	61
L_340_	221	221–248	224	−1.00E-02	63
H_200_	272	272–300	277	−1.16E-02	55
H_220_	271	271–303	276	−1.00E-02	53
H_240_	263	262–280	266	−1.61E-02	55
H_300_	225	225–250	229	−1.00E-02	48

**Figure 6:**
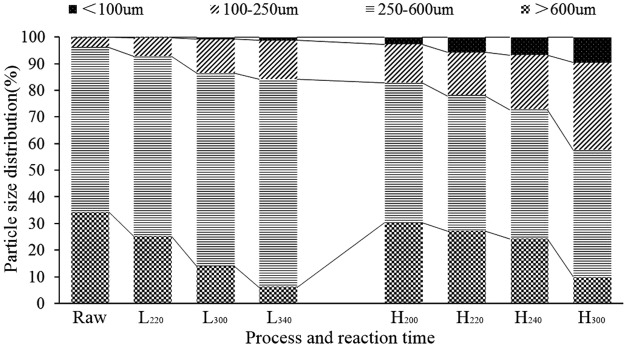
Particle size distributions of selected samples.

It could be deduced that smaller particle size and less moisture content could effectively promote the combustion of the fuel and lower the ignition temperature. Figure 6 shows the particle size distributions of the selected samples. The results indicate that the percentage of smaller size particles (<100 μm, 100–250 μm and 250–600 μm) increased in both processes. In the LTP process, larger particle size (>600  μm) was reduced from 34.27% (Raw) to 6.14% (L_340_). It decreased to 9.95% (H_300_) by the process of HTC. Interestingly, In the range of 100–250 μm, the yields of chars showed an opposite trend which increased from 61.81% (Raw) to 78.11% (L_340_) in LTP while decreased to 47.68% in HTC. It is mainly due to the fact that a large number of particles was received in the range of 0–250 μm by HTC. At the same reaction temperature, the HTC samples showed higher pulverization than those obtained via LTP process. For example, L_300_ produced 12.84% pyrolytic carbons in range of 100–250 μm while the H_300_ produced 32.94% hydrothermal carbon. In the range of less than 100 μm, L_300_ received almost no product while H_300_ had 9.42% hydrothermal carbon. Altogether, through the particle size distribution analysis, it was found that the percentage of smaller size particles increased in both of processes, and it was considered to be one of the main reasons for the decreasing of the ignition temperature.

**Figure 7:**
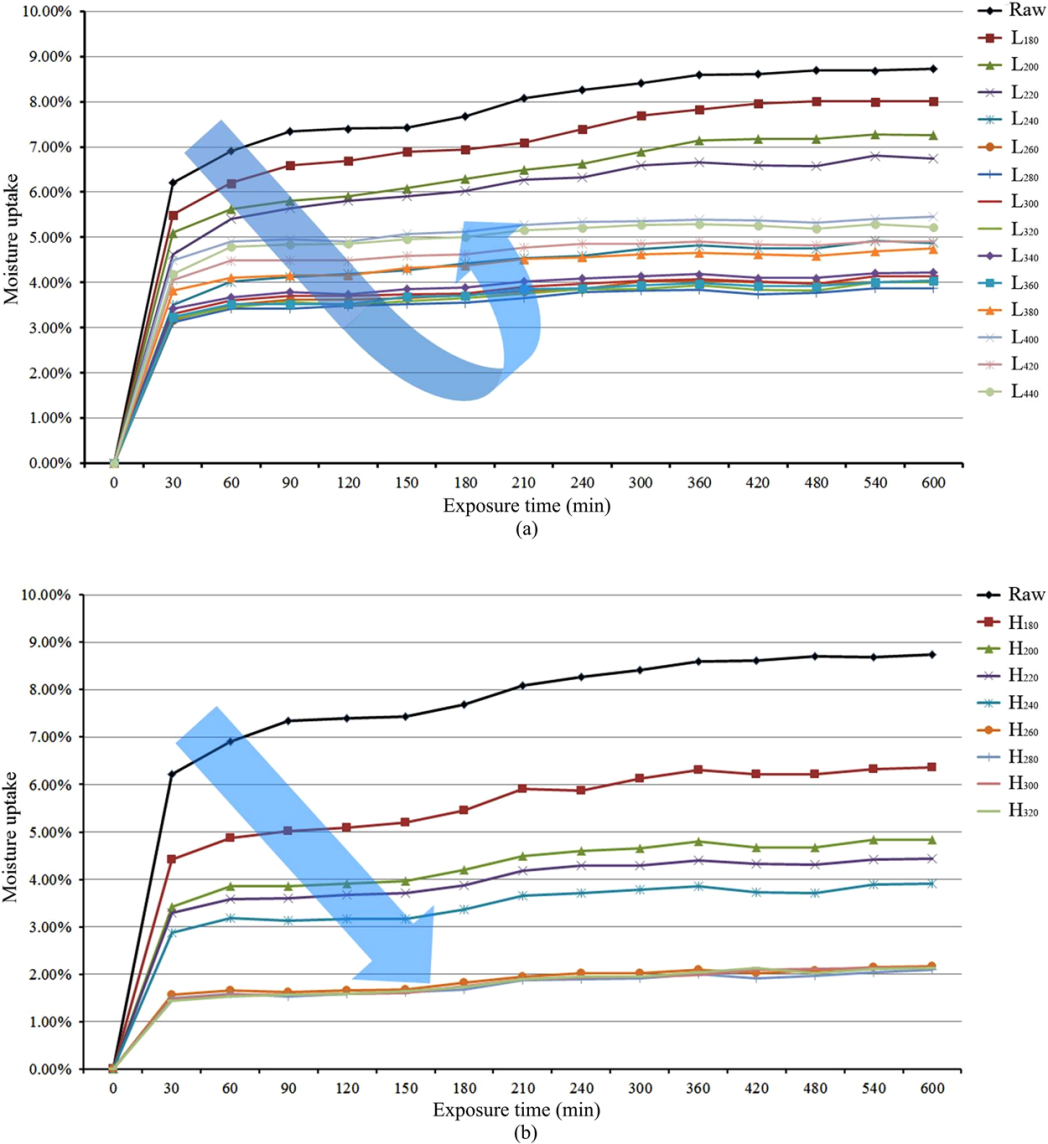
(**a**) Water absorption characteristics of pyrolytic carbon samples. (**b**) Water absorption characteristics of hydrothermal carbon samples.

Moisture resistance is one of the important indicators of biomass fuels. Higher moisture resistance can reduce transportation costs and improve the combustion characteristics of the fuel. Figure 7(a,b) show the moisture uptake profiles of all samples under the storage conditions of 30 °C and 70% relative humidity within 10 h. It is evident that the moisture of the chars reached saturation in 10 h. Compared with the raw material, the treatments of LTP and HTC both improved the moisture resistance of the chars. In Fig. 7(a), it was found that the hydrophobicity of the samples increased with the increase in reaction temperature by LTP before 280 °C. For example, the saturated moisture content of the chars decreased from 8.73 wt.% to 3.88wt.%. Interestingly, this decreasing trend did not continue in the higher temperature region (280 °C–440 °C). Instead, the saturated moisture content was increased to around 5 wt.% (4.90 wt.% for L_420_ and 5.22 wt.% for L_440_). In Fig. 7(b), it was evident that saturated moisture content decreased from 8.73 wt.% to around 2 wt.%. In addition, it is worth mentioning that the hydrophobicity of H_260_, H_280_, H_300_, and H_320_ were quite consistent. It was also found that the change of fixed carbon, volatiles and HHV slowed down. The reason might be that large amount of cellulose and hemicellulose degraded before 260 °C by HTC. Above all, H_260_ is a better choice as a fuel candidate in terms of energy consumption, char yield, fuel rate, and hydrophobicity. Comparing L_200_ and H_200_, it was found that H_200_ had lower saturated moisture content (4.83%) than that of L_200_ (7.26%). Comparing L_300_ and H_300_, it was also found that H_300_ had lower saturated moisture content (2.14% for H_300_, 4.14% for L_300_). Based on the results, under the same reaction temperature, the chars produced by HTC have stronger hydrophobicity than that of the chars prepared by LTP. Therefore, through both the analyses of particle size distribution and moisture resistance, particle size distribution and moisture resistance have more serious effects on the ignition temperature than the degree of carbonization.

Similar to the change of the ignition point, *T*_*max*_ also decreased with the increase in reaction temperature and reduced from 268 °C to 224 °C by LTP. While the *V*_*max*_ had the opposite trend which increased from −1.43E-02%/s to −1.00E-02%/s. With the deepening of reaction degree by LTP process, the temperature interval of the resulting charcoal was increased from 20 °C (261 °C–281 °C for Raw) to 27 °C (250 °C–277 °C for L_340_ and 221 °C–248 °C for L_300_). In selected HTC samples, a similar pattern of *T*_*max*_, *V*_*max*_ and temperature interval was not shown. However, Δ*T* of carbon samples of HTC and LTP showed consistent trends. Temperature differences of pyrolytic carbon samples and hydrothermal carbon samples increased from 45 °C (Raw) to 63 °C (L_340_) and 62 °C (H_300_), respectively. It was considered that Δ*T* has a close relationship with HHV.

Integrated combustion characteristics index (*S*) was used to further evaluate the combustion performance of HTC and LTP in the same reaction temperature. It could be described by the following formula:

6 $$S=\frac{{V}_{\max }\times ({W}_{O}-{W}_{end})}{{T}_{i}^{2}\times {T}_{end}\times t}\times 100 \% $$

where *W*_*O*_ is a corresponding percentage of mass to *T*_0_; *W*_*end*_ is a corresponding percentage of mass to *T*_*end*_; *t* is the time which temperature interval takes. Comparing L_220_ and H_220_, It was found that L_220_ had higher integrated combustion characteristics index (4.11 E-08 wt%^2^ s^−2^ °C^−3^) than that of the H_220_ (1.98 E-08 wt%^2^ s^−2^ °C^−3^) due to the lower *T*_*i*_, *T*_*end*_ and *t*. It showed that compared to H_220_, L_220_ had better combustion performance. However, it showed the opposite result in another set of contrasts including L_300_ and H_300_. L_300_ had a stable integrated combustion characteristics index which was 4.68 E-08wt%^2^ s^−2^ °C^−3^, while H_300_ had a higher result which was 4.95 E-08wt%^2^ s^−2^ °C^−3^ due to the lower *T*_*i*_, *T*_*end*_, and *W*_*end*_ resulting from low ash content. It proved that H_300_ performed better considered only the combustion performance.

## Conclusion

Both HTC and LTP processes are promising carbonization technologies for upgrading the waste wood of *Eucommia ulmoides* Oliver into solid fuels. However, the difference between HTC and LTP is obvious. For the same char yield, the required temperature for HTC is lower than the LTP process. In addition, the HTC process can be carried out without considering the higher moisture content and the hydrothermal carbon samples have the stronger hydrophobicity than that of the chars prepared by LTP. And the H_260_ was selected as a fuel candidate in terms of energy consumption, char yield, fuel rate, and hydrophobicity. In terms of fuel rate, hydrothermal carbon was higher compared with the pyrolytic carbon treated at the same temperature. Through the Fiber analysis and Fourier infrared spectra, it was concluded that the degraded temperature of hemicellulose and cellulose were the critical factor influencing the result of the proximate analysis, char yield and HHV. Compared to the deepening degree of carbonization, particle size distribution and moisture resistance have more significant effects on the ignition temperature.
